# Editorial: Host-microbiota and cancer

**DOI:** 10.3389/fonc.2023.1154586

**Published:** 2023-03-17

**Authors:** Samantha Lopez, Manish K. Tripathi

**Affiliations:** ^1^ Department of Immunology and Microbiology, School of Medicine, the University of Texas Rio Grande Valley, McAllen, TX, United States; ^2^ South Texas Center of Excellence in Cancer Research, School of Medicine, the University of Texas Rio Grande Valley, McAllen, TX, United States

**Keywords:** microbiome, TME, cancer, immunotherapy, colitis

Cancer is the 2^nd^ leading cause of death in the United States, making up an average of 21% of deaths in both genders (1). The COVID-19 pandemic impacted the diagnosis and treatment of cancer, starting from its peak in mid-2020 and still recovering (2). With continued mortality and incidence due to this disease, research has migrated to other areas of interest for mechanisms to aid cancer management, such as exosomes, nucleic acids, and, more innovatively, the gut microbiota. The human microbiome is a complex community composed of various microorganisms, including bacteria, viruses, fungi, and protozoans. It contains approximately 100 trillion microorganisms and can be found at different body points, such as the skin and respiratory system. Still, the majority commonly reside in the gastrointestinal region (3). The connection between microbiome and health began to be established with the microbiome having evolving links in cardiovascular, inflammatory bowel disease, and cancer (4–6). Within the scope of cancer, it is now well known that a high involvement of microbiota can indirectly or directly affect the occurrence, treatment outcome, and drug resistance. For example, *Helicobacter pylori* are cancer-related pathogen that can increase the incidence of gastric cancer (7). This microorganism and others will generally be responsible for approximately 20% of cancer cases (8).

Additionally, the microbiota in the body has been found to have interactions in the tumor microenvironment and has ways to promote or regulate carcinogenesis and cancer therapeutic response. This can occur through signaling pathways, inducing DNA damage, and immune system regulation (9). The ability of the human microbiota to play a functional role in carcinogenesis identifies it as a potential and worthy subject for further research to understand the regulatory mechanisms for pathogen-related cancers. Furthermore, research is essential and beneficial as it can aid in identifying novel therapeutics for cancer management. Studies have shown that the tumor microbiota has a different composition than normal, and the type of composition can affect different points of development and progression.

The microbiome can help regulate cancer at various points and is commonly due to the significant difference in the microbiota composition. Therefore, understanding how the microbiome differs between healthy patients and those with cancer and how pathogenic infection can alter this microbiome is highly important to establish a baseline between the disease and begin identifying potential targets and mechanisms. In a study looking at glioma development, the authors were hoping to determine how glioma could alter the microbiome and, in turn, how the microbiome affected the outcome of glioma tumors (Fan et al.). Using mouse models to establish glioma models, the study was able to report a difference in the microbiome from before tumor development and the one after a tumor was established, showing that glioma tumors influence the composition of the microbiota. On the other hand, when a dysbiosis microbiome was induced through antibiotic treatment, it showed that these altered microbial environments had a worse prognosis in glioma growth, indicating that the microbiome component can interplay with tumor progression.

Additionally, to further cover the study points, after identifying the differences between microorganism composition, they acknowledged specific targets for the microbiome, such as CD8 and Fox3, that can affect tumor growth. A dysbiosis microbiome has not only been found to regulate specific components in glioma tumors specifically but can also play a role in gastric cancer. This topic was discussed in a review covering the role of plasmacytoid dendritic cells (pDC) in gastric cancer (Yang et al.). In gastric cancer, there has been a noticeable alteration in the immune components, such as BDCA2+ pDCs and Foxp3+ Tregs, significantly increasing in tumor vs. normal. As the microbiome also changes in the presence of cancer, there was research to establish if this alteration could be responsible for the immune system changes seen. It was noted that the alterations in bacteria, such as *Stenotrophomonas*, *Selenomonas, Comamonas*, and *Gaiella*, could regulate the abundance of BDCA2+ pDCs and Foxp3+ Tregs. In addition to regulating pCD density, the microbiome was also found to modulate pCD cells themselves, specifically increasing IL-10 secretion to alter immunity. This shows that the microbiome can promote cancer progression through immune system regulation. However, the mechanism behind this alteration remains unclear, with certain studies showing possible routes through cytolytic CD8+T cells. Since the microbiome can regulate tumor growth, it is also essential to study the microbiome to understand its relationship with therapeutics and target the microbiome to help increase efficiency.

The microbiome is a well-regarded environmental influencer meaning it can significantly affect chemical/immunotherapy efficiency and other aspects of treatments. Studying the microbiome in relation to therapies is especially important because it can identify essential bacteria or develop ideas to improve treatments, advance cancer management, and diminish treatment side effects. A current example is a study looking into ICI treatments, more specifically, the monoclonal antibody therapy targeting PDL1/PD1, and establishing a link between the gastrointestinal irAE caused by the treatment and the gut microbiota to better manage irAE. The authors of this study included patients who developed irAE from ICI therapies and patients diagnosed with UC. They were first able to show pathway similarities in ulcerative colitis and irAE, indicating a degree of resemblance in relation to function with the most vital genes that correlated between the two ailments, including ICAM1, ITGB2, and CD86 (Sakai et al.). Their work studying the microbiota between the active forms showed similarities in taxa between the inflamed conditions. They also identified alterations in bacteria, with certain *Bacteroides* species absent and *Enterobacteria* having a higher abundance in active forms of both ailments. The results from this study can be further used in understanding irAE and help understand the pathogenicity of irAE and, therefore, help improve this undesired side effect of ICI immunotherapy. With further research and larger sample sizes, the idea of the microbiota serving a functional role or as a target in improving immunotherapy can be further solidified (Sakai et al.). While this idea is barely coming to large in immunotherapy, there has already been research showing that the microbiome can be targeted to improve current diseases. For example, the microbiome has been studied in relation to antituberculosis therapies and how early targeting of the microbiome can improve treatment for tuberculosis (Diallo et al.). As mentioned, tuberculosis commonly affects the microbiome by causing dysbiosis, which is also common in many types of cancer. An extensive literature review on the gut microbiome and host-directed therapies for tuberculosis brought a range of microbiome-targeted therapies to light that help balance the dysbiosis in tuberculosis, which can be translated to cancer treatment to improve microbial dysfunction. A primary treatment option is correcting the microorganism imbalance using probiotics and prebiotics to provide a productive function to the microbiome, such as inflammation regulation and immune response. Many studies have shown that administering certain strains as a treatment, such as *Lactobacillus johnsonii*, could help with common cancer issues, such as decreasing inflammation and restructuring the microbiome. Additionally, it was further shown that prebiotics and probiotics successfully diminished post-treatment side effects and made therapies more manageable.

Ongoing studies show microbiome plays a more prominent role in cancer progression and could potentially be targeted toward cancer treatment. The research demonstrated how the microbiome is altered in cancer and how this differs from normal and can target a variety of processes, including drug toxicity, treatment effectiveness, and immune response. Future research can identify which mechanisms the microbiome uses to regulate these responses to identify further targets and single out specific microorganisms that can be used to improve cancer treatment and diagnosis.

## Author contributions

MT conceptualized, MT and SL prepared the first draft, finalized and formatted by MT, SL prepared the initial figure, finalized by MT. All authors have read and agreed to the submitted version.
